# Vestibular Loss in Children Affected by LVAS and IP2 Malformation and Operated with Cochlear Implant

**DOI:** 10.3390/audiolres13010013

**Published:** 2023-02-09

**Authors:** Åsa Bonnard, Eva Karltorp, Luca Verrecchia

**Affiliations:** 1 Department of Clinical Science, Intervention and Technology, Division of Ear, Nose and Throat Diseases, Karolinska Institutet, Karolinska University Hospital, Huddinge, B61 141 86 Stockholm, Sweden; 2 Department of Otorhinolaryngology Audiology and Neurotology, Karolinska University Hospital, Huddinge, M53 141 86 Stockholm, Sweden

**Keywords:** LVAS, LVA, IP2, vestibular, vHIT, VEMP, CI, cochlear implant

## Abstract

This is a single center cohort study regarding the prevalence of vestibular loss in hearing impaired children affected by large vestibular aqueduct syndrome (LVAS) with incomplete cochlear partition malformation type II (IP2), fitted with cochlear implant (CI). Twenty-seven children received CI operations at 0.4–13 years on one or both ears and tested for vestibular loss with head impulse test, video head impulse test, mini ice-water test and cervical VEMP. Vestibular loss was found in 19% of operated ears and in 13.9% of non-operated ears. The difference was not statistically significant and was not significantly modified by age at implantation, age at testing, sex, presence of SLC26A4 gene mutation or bilaterality. However, the presence of anatomic anomalies at the level of the vestibulum or semicircular canals was significantly associated with a higher incidence of vestibular loss in CI operated children but not in those non-operated. No other factors, such as the surgical access, the electrode type, the presence of Gusher perioperatively, or post-operative vertigo modified significantly the prevalence of vestibular loss. In conclusion, LVAS/IP2 appears to be the major determinant of vestibular loss in these children, with a less obvious impact of CI, excluding the cases with vestibulum/canal anomalies: this group might have a higher risk for vestibular loss after CI surgery.

## 1. Introduction

Vestibular function is often affected in children with sensorineural hearing loss (SNHL). The prevalence of vestibular loss (VL) in hearing impaired children can reach 50–70% according to different works [1,2]. Children with hearing impairment may exhibit suboptimal levels of postural control, motor skill performance and health-related quality of life [3], and this has been related to a delay in motor milestone acquisition due to vestibular failure.

Inner ear malformation accounts for 20% of childhood sensorineural hearing loss (SNHL) [4], and large vestibular aqueduct (LVAS) with or without incomplete cochlear partition type 2 (IP-2) is the most common malformation, representing approximately 55% of the malformed cochleae [5]. The malformation is seen in Pendred syndrome but can also be non-syndromic and is accompanied by vestibular symptoms in approximately 45% [6]. The inner ear dysfunction in LVAS/IP2 may vary between the two sides in the same subject. The range of hearing loss is from bilateral childhood deafness to a mild to moderate unilateral hearing loss at an adult age, with a progressive nature in approximately one third of cases [7]. The clinical course may vary between the ears. Cochlear implantation is the treatment of choice in LVAS with severe SNHL.

Both inner ear malformations and cochlear implant (CI) are associated with VL [8]. Inner ear malformations are, per se, a risk factor for vestibular dysfunction, with up to 50% of affected children showing vestibular impairment [9]. The grade of vestibular malformation seems to be related to the grade of vestibular impairment and the motor developmental delays [10]. Moreover, a late development of vestibular reflexes appears to be associated to motor delays in inner ear malformed children [11].

The detrimental effect of CI on vestibular function and on motor performance was reported by some authors [12,13]. Comparative studies have shown that among the vestibular tests, the vestibular evoked myogenic potentials (VEMP) resulted in children that were more affected by CI implantation [14], and a VEMP deficit was shown to predict motor proficiency delays in CI children [15]. However, the association between CI and VL is still questioned, as other authors have not observed the same unfavorable effect of CI on vestibular function [16].

Taken together, whether CI is a safe intervention for vestibular function in children, and especially in children with pre-existing vestibular impairment associated with inner ear malformations, is still a matter of discussion and worth scientific investigation.

Here, we present a descriptive analysis of a cohort of children with LVAS/IP2, all habilitated with CI and regularly followed at our tertiary national referral center for treatment of childhood deafness with malformed ears. The study aimed to define the prevalence of vestibular loss in LVAS/IP2 and the safety of CI surgery on vestibular function. These two outcomes were weighted on different clinical characteristics related to the subject, disease and surgery.

## 2. Materials and Methods

### 2.1. Patients

All children with LVAS with or without IP2 malformation who received operations at the CI center at our tertiary referral hospital between 1991 and 2017 were invited to participate in the study. A follow-up of at least 6 months at the day of examination was required for inclusion. The children without LVAS or cochlear malformation, other types of malformation or having a follow-up of less than 6 months were excluded. The study was performed in accordance with the Declaration of Helsinki guidelines and obtained the ethical approval from the regional board of ethics Nb 2015/1296-31/2. All parents or adult participants gave their written informed consent to inclusion. The project was hindered by the COVID-19 epidemic with a consistent delay in data sampling and analysis. At last, 38 patients were identified and 29 agreed to participate (76%). Of the nine patients choosing not to participate two were CI non-users. The vestibular function could be tested in 27/29 children and information about preoperative vestibular function could be retrieve in 7 of those 27 children.

Study population characteristics and clinical data were collected retrospectively from clinical notes. Beyond gender and age at testing, the age at first and second surgery and the operated side were retrieved. Moreover, the results of genetic testing for SLC26A4 mutation and the extension of inner ear malformation (aqueduct, cochlea, canal/vestibulum) as shown in radiological examination were retrieved. Some surgical data were also taken into account: type of access (round window/cochleostomy), type of electrode (perimodiolar or lateral wall), perioperative gusher and postoperative vertigo. A few children could be tested also before the first and/or the second implantation; in these cases the testing was performed from days to 1–2 weeks before intervention. Demographics and the above-mentioned clinical data are listed in Table 1. Given the retrospective sampling, missing data was present, and analysis was conducted on the available data. 

### 2.2. Method

#### 2.2.1. Radiological Exam

A preoperative CT and/or MRI was performed in all children. The Cincinnati criteria for LVA was used with >0.9 mm in the midpoint of the vestibular aqueduct and/or >1.9 mm at the operculum [17]. For the IP2, the Sennaroglu classification from Sennaroglu et al. 2002 was applied [4].

#### 2.2.2. Radiological Exam

All surgeries were performed with a slow, atraumatic approach through the round window or a cochleostomy with regard to the surgeon’s preference and/or the suggested method from the companies at the time of surgery. Before 2010, cochleostomy was the general method of choice at the unit regardless of electrode and implant company. In case of oozing after the opening of the scala tympani, the implantation was performed after the oozing had ceased. The implant was tested before wound closure and all children had a postoperative radiological exam.

#### 2.2.3. Vestibular Investigation

Child-friendly vestibular testing has been in use at our tertiary center since 2015. The method is described in details in a previous validation study [18]. It consists of:

1. Head impulse test (HIT): HIT tests the integrity of the angular vestibular ocular reflex (aVOR) in stabilizing the gaze during passive jerk head rotations in the latera plan. After locking the child’s attention on a fixation point, the operator observed the child´s gaze during repetitive jerk head rotations. Visible gaze refixations could be reproduced in rotations towards the ear affected by VL. HIT was defined pathological (indicative of VL) when gaze refixations could be reproduced in at least three head turns on the same side.
2. Video-Head Impulse Test (vHIT): it is a video-assisted version of the HIT in which the VOR deficit could be quantified in terms of eye/head velocity gain during head jerk rotations under visual fixation in the lateral plan. Using the Synapsys Video Head Impulse Test Unit (Ulmer version 2), a VL was defined when the gain, calculated on at least 3 rotations per side, resulted lower than 0.7 [19].
3. The caloric test was conducted according to a modified mini ice-water test technique [20]. VL was defined by the absence of a caloric nystagmus in response to a minimal ice-water ear irrigation (ear canal filled with water at 6–10 °C, for 10 s); caloric nystagmus was defined as two cluster of 3 or more nystagmus beats toward the not irrigated side at videooculoscopy, separated by a short visual fixation interval.
4. Cervical Vestibular evoked myogenic potentials (cVEMP): The VEMP was evoked by mastoid bone conducted stimulation. Stimuli were delivered with a Radioear SR71 transducer, 500 Hz tone burst at 50 dB HL (125 dB Pe SPL) and responses recorded at the ipsilateral sternocleidomastoideus with surface electromyography (EMG), by a Interacustic eClipse Averager. One hundred and twenty sweeps were collected for each recording, in the condition of muscle activity steadily maintained between 50–400 μVolts and obtained by the head rotated to the opposite side. A VEMP response was identified as a short latency positive–negative EMG deflection, with a positive peak at 11–17 ms and a negative peak at 19–25 ms after stimuli. The amplitude in μVolts was corrected for background EMG (scaled amplitude), given the linear association between the two. The responses were identified morphologically, based on two principles: the presence of an identifiable P-N deflection and, in case of noisy recordings, a least scaled amplitude of 0.18, according to our previous study [21]. Finally, the absence of identifiable VEMP responses in three consecutive trials was taken as a marker of VL.

By this vestibular assessment, it was possible: a. to test all children in a large age span with the same procedure; b. to test the two ears separately for VL and c. obtain a vestibular response from at least one of the four different test modalities, according to age related differences in tests’ compliance. The subjects were asked to switch off or remove the outer processor during testing. The four vestibular tests appraise different input/output functions of the unilateral vestibular activity, and a certain grade of test discordance is expected [22]. The HIT and vHIT tested the (lateral) semicircular ocular reflex at high frequency range and under visual fixation; the mini ice-water test investigated the same semicircular canal function at low frequency range and without visual fixation. The cVEMP, as described here, was used as a measure of the sacculo-collic reflex, and more in general, of otolith function. We have maintained HIT even in the presence of vHIT, because vHIT was difficult to perform in some children (intolerance for head restraining, invasive blinking reflex). On the other hand, the HIT could be falsely negative in the presence of cover saccades (not visible at HIT). These cases could be correctly identified by vHIT. In the referred validation study, test concordance and compliance were high between the canal tests (caloric irrigation, HIT and vHIT), with at least one canal test valid for each tested subject. The otolith test (VEMP) showed a lower concordance with canal tests and lower compliance in general. For this reason, the data were analyzed not only in terms of VL (both canal and otolith loss) but also as selective canal loss and selective otolith loss.

### 2.3. Data Analysis

Having a method able to test for unilateral vestibular loss and assuming that the two sides in the same subject may be considered independently regarding the clinical course, the dataset was restructured focusing on affected ears and not on affected subjects. We obtained three groups of interest: 42 operated ears, 22 not operated ears (ears tested before planned CI or second non-operated ears in unilaterally implanted) and 10 ears (in seven children) tested before and after surgery. Vestibular test results and clinical characteristics were studied on these three groups. Accordingly, the study aimed:

(1) To ascertain whether the vestibular loss in ears affected by LVAS and operated with CI had a different prevalence than in LVAS affected, but not operated, ears.
(2) To define possible clinical characteristics affecting the incidence of vestibular loss in CI operated vs not operated ears.

#### Statistical Analysis

The analysis was conducted with non-parametric tests (Fisher’s exact test) for the crude prevalence of vestibular loss in not operated vs operated ears. Only a descriptive analysis could be conducted in the 10 ears that were tested before and after CI operation. The association between the clinical characteristics and vestibular loss in operated/non operated ears was analyzed with Fisher’s exact test. Mann–Whitney U test was used for the study of vHIT gain and corrected amplitude, P1 and N1 latencies in VEMP comparing the values obtained in non-operated and operated ears. The same analysis was conducted on vHIT and VEMP amplitude weighted on the different clinical characteristics. The level of statistical significance was stated at α < 0.05.

## 3. Results

### 3.1. Clinical Characteristics

Of 27 children, 15 received bilateral cochlea implants and 12 unilateral CI (bimodal amplification). Additionally, 14/27 (52%) of operated children were females. The first CI was implanted at a median age of 1.9 yrs (IQR: 1.6, min: 0.4; max: 6.1), the second CI was implanted at a mean age of 4.7 yrs (SD: 4.1, min: 0.7; max: 13.3). Children were tested at a mean age of 9.8 yrs (SD: 6.1; min: 1.4; max: 25.2) with a median time interval between the last CI operation and vestibular testing of 2.7 years (IQR: 6.6; min: 0.5; max: 23).

**Figure 1:**
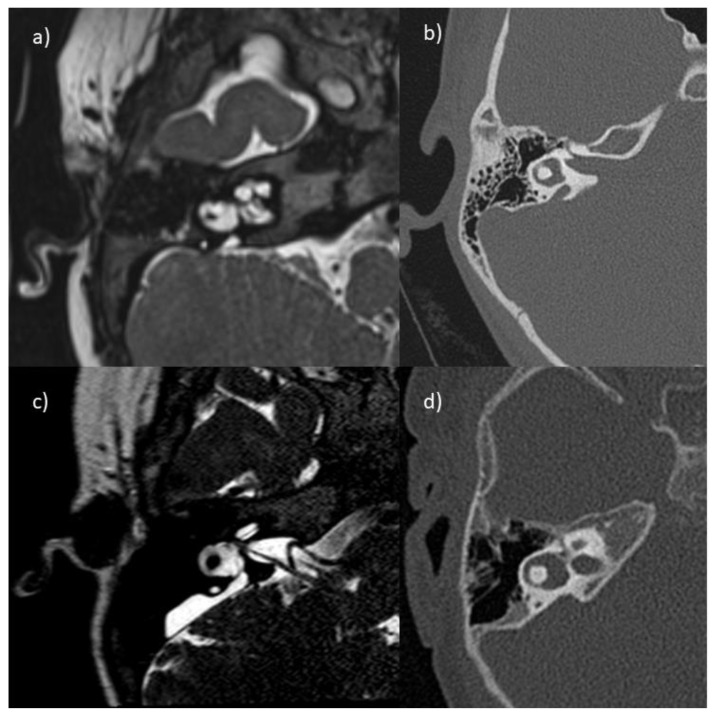
Images of right temporal bone in four children showing examples of dilated lateral semicircular canals at the ampulla portion (**a**–**d**) and a displacement of the bony center with a narrowing of the canal (**a**). Magnetic resonance imaging in (**a**,**c**), and computer tomography in (**b**,**d**).

LVAS malformation was present bilaterally in all but one child (unilateral LVAS). Additionally, 24 (89%) had an associated bilateral IP2 malformation. Only five children showed a bilateral canal/vestibulum malformation, four in the presence of both LVAS and IP2 counterparts and one with only LVAS (Figure 1). The operated ears with anomalies were 8, the ones without were 31 (missing data in 3). Out of 23 children tested for the genetic mutation SLC26A4, 17 (74%) had positive results for the mutation.

Of the 42 operated ears, 13 were implanted through a cochleostomy and 29 with a round window approach. The round window approach prevailed in both the bilateral operations and in unilateral CI surgery. The bilateral surgeries were not conducted simultaneously in any child. A modest gusher or oozing was noted in 20/29 (69%) surgical reports.

Specific information on post-operative vertigo was reported in the medical notes related to 38 ears. Four out of these had important dizziness/vertigo after surgery. The balance normalized in all at the outpatient control at 3–4 weeks post-surgery.

### 3.2. Vestibular Testing

#### 3.2.1. Compliance and Test Concordance

In total, 64 vestibular testing procedures were conducted, 22 in non-operated ears and 42 in operated ears. HIT had valid responses in 63 of them, vHIT in 58 and caloric test in 47. At least one canal test could be completed in all cases. In 50/64 (78%), a VEMP test could be completed with valid results. Accordingly, the most feasible test was the HIT, the least the caloric stimulation. In seven procedures, the canal tests resulted as discordant: in four, the vHIT/HIT showed a normal response, with caloric irrigation indicating VL. In the other three cases, the vHIT/caloric irrigation resulted as discordant from HIT. The vHIT response was deemed as the most reliable and used as a reference.

A canal/otolith discordance was found in 3/64 tests: a selective canal loss (with normal VEMP response) was found in 1 case and a selective otolith loss (with normal canal responses) in 2 others. These three cases were all found in operated ears and interpreted as a residual vestibular function, and thus, not included in the VL count.

#### 3.2.2. VL and CI

The 22 non-operated ears showed VL in 3 (13.6%) and the 42 CI operated ears in 8 (19%). Individual data are listed in Table 2. The difference was not statistically significant. Of 10 ears tested both before and after the operation, one showed VL preoperatively (10%) that improved postoperatively, and one functioned normally preoperatively, showing VL postoperatively (10%). Taken together, it was an equal distribution of VL prevalence before and after operation.

Canal loss was present in 3/22 (13.6%) of non-operated ears and in 9/42 (21.4%) of the operated ones the difference was not statistically significant. Loss in otolith function was retrieved in 13.3% (2/15) of non-operated ears and in 24.2% (8/33) of operated ears, with no statistical difference. A parametric analysis conducted on vHIT (gain) and VEMP values (corrected amplitude, P1 latency and N1 latency) showed no statistically significant differences between non-operated vs operated ears (Table 3).

Among the bilaterally operated children, the prevalence of VL did not differ between the first operated and the second operated ears. The prevalence of VL was not statistically different in ears bilaterally operated compared with unilaterally operated ears —2/12 (6.7%)— vs. —6/30 (20%)— ears operated bilaterally.

In two bilateral cases, the vestibular test was also conducted between the two operations because of post operative vertigo/vomiting after the first operation. Both of them showed a VL on the operated side. The operation on the second side resulted in a bilateral VL in one case and a unilaterally preserved function for the other.

#### 3.2.3. Clinical Characteristics in Operated and Non-Operated Ears

The prevalence of VL in operated ears was not significantly altered by any considered clinical characteristic but one: the presence of canal/vestibulum anomalies. Operated ears with added anomalies showed a VL proportion—4/8 (50%)—significantly higher than operated ears without added anomalies—4/31 (12.9%)—(*p* = 0.040). The same result was found comparing both operated and non-operated ears with and without added anomalies: there was a statistically significant difference between the four binomial proportions (Fisher’s exact test of homogeneity (2 × 4), *p* = 0.048); however, the only one that resulted statistically significant at pair comparisons was the above mentioned, i.e., operated ears with added anomalies vs. operated ears without added anomalies. According to this analysis, the combination of CI operation in presence of canal/vestibular anomalies may increase the risk to develop VL in LVAS/IP2 ears. Interestingly, of the two bilaterally operated children showing a unilateral VL at an intermediate testing, both had canal/vestibulum anomalies. In line with the above-mentioned analysis, one out of two (50%) showed injured vestibular function also on the second operated ear.

A weak association was also found between electrode type and VL, with 3/7 ears operated with a perimodiolar electrode showing VL (42.9%) in comparison to the 3/33 (9.1%) with a lateral wall electrode (*p* = 0.055). Similarly, the vHIT gain was significantly reduced in ears operated with a perimodiolar electrode compared with a lateral wall electrode: 0.73 (0.24) vs. 0.98 (0.15), *p* = 0.033 (Table 4). However, these relations were biased by the association between the electrode type and the presence of canal/vestibulum anomalies in ears with perimodiolar electrode —4/6 (67%—) vs. in ears with lateral wall electrode—5/32 (15.6%)—(*p* = 0.02). 

Otherwise, the VL prevalence was not significantly different when weighted on age at intervention, gender, post-operative vertigo, gusher, CI model, surgical approach, presence of SLC26A4 mutation and bilateral/unilateral surgery.

## 4. Discussion

Vestibular loss, especially the bilateral form, is one of the major determinants of motor abilities in SNHL-affected children, as supported by accumulated clinical evidence [8,23]. The severity of SNHL seems to correlate with vestibular impairment [24]. Vestibular assessment is conducted routinely in CI centers, both in order to appraise the residual vestibular function before CI but also to check for possible vestibular injuries after CI surgery.

In this cohort of LVAS/IP2 children, we have found a non-negligible prevalence of VL loss among the tested ears. In CI operated ears, the prevalence of VL was approximately 19%, less in non-operated ears (13.6%). The difference was, however, not statistically significant. By these data it would seem that LVAS/IP2 themselves were the major determinant of VL, making the CI surgery a relatively safe procedure in terms of vestibular injury. VL appeared to be independent also from most clinical characteristics, namely, age, gender, the presence of SLC26A4 mutation, the surgical approach, electrode type, perioperative gusher or post operative vertigo. The only significant relevant factor was the radiological evidence of an added vestibulum or canal anomaly, in addition to the LVAS/IP2 malformation. The presence of those anomalies increased significantly the proportion of operated ears with VL in comparison to both non-operated and operated ears without added anomalies. If confirmed by larger studies, this parameter may represent a risk factor for vestibular injuries in ears planned for CI operation.

LVAS/IP2 are a recognized source of vestibular dysfunction: according to Zalewski et al., up to 45% of the 106 patients with LVAS reported vestibular symptoms whose severity was related to the bilaterality and presence of a head trauma but without apparent correlation with the hearing loss. Markedly, a vestibular dysfunction could be measured in only one third of the ears tested with caloric irrigation [6]. Conversely, the majority (89%) of the 77 LVAS children (3–12 years) tested by Yang et al. [25] with a comprehensive vestibular battery (cVEMP, posturography, rotational chair, caloric testing) showed an abnormality in at least one test, with the lowest incidence at VEMP (15%) and the highest at posturography (69%). However, vertigo was reported by only 22% of subjects and a delayed independent walking age in approximately 37%. In another series of 18 older children–adults presented by Zhou et al. [26], the authors could demonstrate a high grade of abnormal results at caloric irrigation (87.6%), whereas the VEMP showed generally preserved results with enhanced amplitude and threshold. A clear role of CI surgery in the modification of vestibular function in LVAS patients cannot be drawn by these references. However, in a recent series of LVAS affected children, the authors showed quite intact vHIT measures before and after the CI operation, whereas the caloric test and VEMP testing were affected in one third and in one fourth, respectively, of the sample, with a significant worsening after surgery [27]. According to our sample, the prevalence of VL was approximately 14% in non-operated ears and 19% in CI operated ones. Our results are overall less concerning than other contributions, supporting that CI surgery is a safe procedure in children with LVAS/IP2 in terms of vestibular injury. However, this discrepancy in the literature may reside in the different test methods, taking into account that the test protocol applied here was not aimed to grade the vestibular function but rather to identify the unilateral CL in a large age span of children, with varying compliance to vestibular procedures. In general, there is a lack of a harmonization of test methods [28] with the use of different test procedures, different outcomes and different population samples, which makes it hard to draw generalizable conclusions.

It is also worth mentioning that the clinical course in LVAS/IP2 is often characterized by functional fluctuations both regarding hearing and balance, with only a third of the studied cases showing a progressive worsening [29]. Vertigo and dizziness spells are a common complaint of LVAS/IP2 patients, probably an expression of vestibular fluctuation or stepwise degradation [6]. This aspect should be taken into account when interpreting vestibular testing, especially in singular measures over time. For example, is the singular case of post-surgical vestibular reactivation in our sample related to CI stimulation or to a vestibular fluctuation?

The VL prevalence in children operated bilaterally (6/30 ears) is not significantly different from the one in unilaterally operated children (2/12 ears). However, in one case, the operation on the second ear completed a bilateral loss (in the presence of vestibulum/canal anomalies). By these data, bilateral CI surgery seems, overall, a viable option for children with LVAS/IP2, with a marginal risk for bilateral vestibular failure, as anecdotally shown in our data.

## 5. Conclusions

The LVAS/IP2 malformation is, per se, a condition predisposed to vestibular loss. CI surgery appears, by our data, to be a relatively safe procedure in terms of vestibular injury. Interestingly, the presence of added anatomical anomalies at vestibulum or semicircular canals may represent a risk factor for CI related vestibular injuries. If these results are confirmed by larger studies, CI surgery may be planned in LVAS/IP2, taking into account the presence of these anomalies.

## 6. Limitations

The slight difference in vestibular loss proportion in operated ears compared to non-operated ones (+5.4%) may be addressed to an underpowered sample for this analysis, together with the heterogeneity of the cohort in terms of associated covariables (for ex., surgical technique, monolateral vs. bilateral sequential surgery, different interval between surgeries). The same observation may be done, however, for other published works on the same issue. LVAS/IP2 is a rare condition and heterogeneous in clinical presentation with difficulties in obtaining a robust clinical dataset. This study, however, has permitted us to define the prevalence of vestibular loss in LVAS/IP2 ears and to discuss/promote a possible marker of CI-associated vestibular loss: the presence of an added vestibulum/canal anomaly.

## Figures and Tables

**Table 1:** Demographics and clinical characteristics. I/II CI: first and second cochlear implants. Ages expressed in years; positive/negative at test: 1/0; NR: not retrieved. -: not tested. SLC26A4: genetic mutation for gene SLC26A4. Other: vestibulum/canal anomalies. Access: c = cochleostomy, rw = round window. Electrode, lateral = lateral wall; peri = perimodiolar. Vertigo: post operative vertigo. ^†^: one subject showed IP2 on one side, thus, was counted as 0.5.

Subject Factors			Disease Factors				Surgical Factors						
#	Age at Testing	Sex	SLC26A4	LVAS	IP2	Other	Op. Side	Age I CI	Age II CI	Access	Gusher	Electrode	Vertigo
1	18.6	♂	1	1	1	0	right	2.2		C	1	Lateral	0
							left		6.3	C	0	Lateral	0
2	11.8	♂	1	1	1	0	right		2.5	C	1	Lateral	0
							left	2.0		Rw	1	Lateral	0
3	4.3	♀	1	1	1	1	right		3.8	Rw	1	Peri	0
							left	3.1		Rw	NR	Lateral	0
4	6.9	♀	0	1	0	NR	-			-	-	-	-
							left	4.7		Rw	0	Lateral	0
5	12.1	♀	1	1	1	0	right		2.3	C	NR	Lateral	NR
							left	1.9		Rw	NR	Lateral	NR
6	14.3	♂	1	1	1	0	right	2.5		C	1	Lateral	0
							left		11	Rw	NR	Lateral	1
7	2.2	♀	1	1	1	1	right	1.0		Rw	1	Lateral	0
							-			-	-	-	-
8	8.7	♂	1	1	1	0	right		1.4	Rw	1	Lateral	0
							left	0.8		Rw	1	Lateral	0
9	15.3	♀	1	1	1	0	right	2.4		C	1	Lateral	0
							left		3.6	C	0	Lateral	0
10	9.4	♀	0	1	1	0	right	5.2		Rw	0	Lateral	0
							-			-	-	-	-
11	22.9	♂	1	1	1	0	right	3.8		C	1	Lateral	1
							-			-	-	-	-
12	2.8	♂	0	1	0	1	right		2.2	Rw	0	Lateral	0
							left	1.1		Rw	NR	Lateral	-
13	15.3	♀	NR	1	1	0	right	5.1		Rw	NR	Lateral	0
							left		13.3	Rw	NR	Lateral	1
14	6.1	♀	NR	1	1	0	right	0.8		Rw	NR	Lateral	0
							left		1.5	Rw	NR	Lateral	0
15	10.9	♀	1	1	1	0	right		8.3	Rw	1	Lateral	0
							left	6.1		Rw	1	Lateral	0
16	3.1	♀	1	1	1	0	right	0.4		Rw	1	Lateral	0
							left		0.7	Rw	1	Lateral	0
17	10.2	♂	1	1	1	0	-			-	-	-	-
							left	7.5		C	0	NR	0
18	10.8	♂	1	1	1	0	right	1.8		NR	NR	Peri	NR
							left		10.7	C	0	Peri	0
19	11.9	♂	1	1	1	NR	-			-	-	-	-
							left	5.8		C	1	Lateral	0
20	4.4	♂	1	1	1	0	right	1.6		Rw	1	Lateral	0
							left		1.9	Rw	0	Lateral	0
21	7.8	♂	1	1	1	0	-			-	-	-	-
							left	4.9		Rw	1	Lateral	0
22	3.9	♀	NR	1	1	0	-			-	-	-	-
							left	3.3		Rw	1	Lateral	-
23	1.8	♂	NR	1	1	1	right		1.2	Rw	1	Peri	0
							left	0.9		Rw	NR	Peri	0
24	25.2	♀	1	1	1	0	right	2.2		C	1	NR	0
							left			-	-	NR	-
25	13.0	♀	0	1	1	1	right	12.6		Rw	NR	Peri	1
							-			-	-	-	-
26	6.3	♂	0	left	0	0	-			-	-	-	-
							left	1.1		Rw	0	Lateral	0
27	6.4	♀	0	1	1	NR	right	5.8		Rw	NR	Lateral	0
							-			-	NR	Lateral	-
Sum	9.8(6.1)	♀ 14 ♂ 13	17/23	26.5 ^†^/27	24/27	5/24	L22 R20	1.9 (1.6)	4.7 (4.1)	Rw 29 C 41	20/29	Lat 36 Peri 41	4/37

**Table 2:** Results of vestibular testing for subjects and ears. Data relative to operated and non-operated conditions. When results appear in both columns for the same ear, it means that the ear has been tested before and after surgery. 1/0: tested and showing vestibular response (1) or vestibular loss (0); -: not performed; NC: not completed. Vest: vestibular function (at least on one test modality) as result of available test responses.

# Subject	Side	Non-Operated Ears					Operated Ears				
		HIT	vHIT	Caloric	VEMP	Vest	HIT	vHIT	Caloric	VEMP	Vest
1	right	-	-	-	-	-	1	1	1	1	1
	left	-	-	-	-	-	1	1	1	NC	1
2	right	-	-	-	-	-	1	1	1	1	1
	left	-	-	-	-	-	1	1	1	1	1
3	right	1	1	1	1	1	1	1	1	1	1
	left	1	1	1	1	1	1	1	1	1	1
4	right	1	NC	NC	NC	1	-	-	-	-	-
	left	1	NC	NC	NC	1	1	1	1	1	1
5	right	-	-	-	-	-	1	1	0	1	1
	left	-	-	-	-	-	1	1	1	1	1
6	right	-	-	-	-	-	1	1	1	1	1
	left	-	-	-	-	-	1	1	1	1	1
7	right	-	-	-	-	-	1	1	1	NC	1
	left	1	1	NC	NC	1	-	-	-	-	-
8	right	-	-	-	-	-	1	1	1	1	1
	left	-	-	-	-	-	1	0	0	0	0
9	right	-	-	-	-	-	1	1	1	1	1
	left	-	-	-	-	-	1	1	1	0	1
10	right	-	-	-	-	-	1	1	1	1	1
	left	1	1	1	1	1	-	-	-	-	-
11	right	-	-	-	-	-	0	0	0	1	1
	left	1	1	1	1	1	-	-	-	-	-
12	right	0	1	1	1	1	1	1	NC	NC	1
	left	0	1	0	1	1	NC	0	NC	0	0
13	right	-	-	-	-	-	1	1	1	1	1
	left	-	-	-	-	-	1	1	1	1	1
14	right	-	-	-	-	-	1	1	1	1	1
	left	-	-	-	-	-	0	0	0	0	0
15	right	-	-	-	-	-	1	1	1	1	1
	left	-	-	-	-	-	1	1	1	1	1
16	right	-	-	-	-	-	0	NC	0	NC	0
	left	-	-	-	-	-	1	1	1	NC	1
17	right	1	1	1	1	1					
	left	-	-	-	-	-	0	0	0	0	0
18	right	-	-	-	-	-	1	1	NC	NC	1
	left	-	-	-	-	-	1	1	NC	NC	1
19	right	1	1	NC	NC	1	-	-	-	-	-
	left	-	-	-	-	-	1	1	NC	NC	1
20	right	1	NC	NC	1	1	1	1	1	1	1
	left	1	NC	NC	1	1	1	1	1	1	1
21	right	1	1	1	1	1	-	-	-	-	-
	left	0	0	NC	NC	0	1	NC	1	1	1
22	right	1	1	NC	1	1	-	-	-	-	-
	left	1	1	NC	NC	1	1	1	NC	1	1
23	right	-	-	-	-	-	0	0	0	0	0
	left	-	-	-	-	-	0	0	0	0	0
24	right	-	-	-	-	-	1	1	0	0	1
	left	0	0	0	0	0	-	-	-	-	-
25	right	-	-	-	-	-	0	0	0	NC	0
	left	1	1	1	NC	1	-	-	-	-	-
26	right	0	0	0	0	0	-	-	-	-	-
	left	-	-	-	-	-	1	1	1	1	1
27	right	1	1	NC	1	1	1	1	1	1	1
	left	1	1	NC	1	1	-	-	-	-	-

**Table 3:** Parametric analysis of vHIT and VEMP measures in operated and non-operated ears.

		Non-Operated Ears	Operated Ears	Mann–Whitney (*p* Value)
vHIT	GAIN	0.89 (0.11)	0.84 (0.32)	271 (*p* = 0.134)
VEMP	Corr. amplitude	0.52 (0.60)	0.26 (0.26)	182 (*p* = 0.143)
	P1 latency (ms)	14.2 (1.1)	13.5 (1.4)	103 (*p* = 0.069)
	N1 latency (ms)	21.5 (2.2)	22.4 (2.0)	112 (*p* = 0.119)

**Table 4:** vHIT gain and VEMP amplitude values in non-operated and operated ears. Within the operated ears, comparisons weighted on the different clinical characteristics are presented. Significant associations are shown (* = *p* < 0.05).

		vHIT		VEMP
Ears		gain		Corr. amplitude
Non-operated		0.84 (0.32)		0.27 (0.27)
Operated		0.89 (0.11)		0.52 (0.60)
Operated ears:				
SLC26A4	Positive	0.93 (0.23)		0.31 (0.30)
	Negative	0.80 (0.34)		0.26 (0.24)
Anomalies	Present	0.63 (0.43)		0.13 (0.19)
	Absent	0.93 (0.22)		0.28 (0.27)
Access	Round w.	0.86 (0.29)		0.28 (0.26)
	Cochleost.	0.90 (0.30)		0.23 (0.28)
Gusher	Negative	0.94 (0.16)		0.14 (0.12)
	Positive	0.88 (0.35)		0.30 (0.28)
Electrode	Lateral wall	0.98 (0.15)	] *	0.28 (0.25)
	Perimodiolar	0.73 (0.40)		0.10 (0.09)
Postop	Vertigo	0.54 (0.56)		0.29 (0.08)
	No vertigo	0.90 (0.24)		0.25 (0.29)

## Data Availability

The dataset can be provided by direct contact with the corresponding author.
